# Genome Sequence of *Bacillus glycinifermentans* TH008, Isolated from Ohio Soil

**DOI:** 10.1128/genomeA.01573-15

**Published:** 2016-01-21

**Authors:** Daniel R. Zeigler

**Affiliations:** Bacillus Genetic Stock Center, The Ohio State University, Columbus, Ohio, USA

## Abstract

The genome sequence of an Ohio soil isolate, TH008, was determined. The sequence reveals a close relationship between TH008 and domesticated *Bacillus glycinifermentans* strains found in a traditional Korean fermented soybean food.

## GENOME ANNOUNCEMENT

Recently, there has been a burgeoning interest in traditional fermented foods for their potential health and other societal benefits (1, 2). One such food is the Korean fermented soybean paste, cheonggukjang. Two spore-forming bacterial isolates from this food were found to comprise a novel species denoted *Bacillus glycinifermentans* (3). Interestingly, a survey of Ohio soil bacteria capable of growing at 55°C in NaCl 5% (wt/vol) revealed a relatively high frequency of isolates that clustered with *B. glycinifermentans* in multilocus sequence typing analysis (not shown). To determine whether these isolates could be considered “wild” relatives of domesticated *B. glycinifermentans* strains, one typical isolate, strain TH008, was selected for analysis. TH008 had been isolated from a moist soil sample collected on the banks of the Olentangy River in Columbus, Ohio (40.0024 N, 83.0219 W).

To determine the genome sequence for TH008, an Illumina paired-end library (2 × 150 bp) was sequenced. Prior to assembly, 4 million read pairs were selected and sequence adapter trimming was performed followed by digital normalization (4). *De novo* assembly was performed on the resulting normalized paired-end reads with the Velvet assembler (5), using a minimum contig length cutoff of 1,000 bp and k-mer length 77. The assembly had 111 contigs with coverage 39.0; the *N*_50_ was 97,587, and the largest contig was 225,240, and the total size was 4.459 Mb. Genome annotation was performed by the NCBI Prokaryotic Genome Annotation Pipeline (http://www.ncbi.nlm.nih.gov/genome/annotation_prok/). Clusters predicted to encode secondary metabolites were identified by antiSMASH 3.0 (6). A total of 4,522 genes were predicted, including 4,294 coding sequences (CDS), 241 pseudo genes, 41 tRNAs, 5 rRNA genes, and 114 frameshifted genes. The average DNA G+C content was 46.1%.

*In silico* DNA-DNA hybridization (7) with formula 2 of the GGDH 2.0 online application (http://ggdc.dsmz.de/distcalc2.php) estimated the hybridization of TH008 with the known cheonggukjang isolates of *B. glycinifermentans*, GO-13^T^ (GenBank LECW01) and KJ-17 (GenBank LECV01) as 67.60% ± 2.91 and 67.20% ± 2.91, respectively. This result indicates a high likelihood (73.57% and 72.63%, respectively) that TH008 shares a common species identity with these isolates. Ohio soils therefore appear to contain bacteria closely related to flora found in a Korean fermented food.

### Nucleotide sequence accession numbers.

This whole-genome shotgun project has been deposited in DDBJ/ENA/GenBank under the accession no. JZBT00000000. The version described in this paper is the first version, JZBT01000000.
